# Reinforced tension-line suture after laparotomy: early results of the Rein4CeTo1 randomized clinical trial

**DOI:** 10.1093/bjs/znae265

**Published:** 2024-10-30

**Authors:** Charlotta L Wenzelberg, Peder Rogmark, Olle Ekberg, Ulf Petersson

**Affiliations:** Department of Clinical Sciences Malmö, Lund University, Lund, Sweden; Department of Surgery, Skåne University Hospital Malmö, Malmö, Sweden; Department of Clinical Sciences Malmö, Lund University, Lund, Sweden; Department of Surgery, Skåne University Hospital Malmö, Malmö, Sweden; Department of Translational Medicine Malmö, Lund University, Lund, Sweden; Department of Radiology Diagnostics, Skåne University Hospital Malmö, Malmö, Sweden; Department of Clinical Sciences Malmö, Lund University, Lund, Sweden; Department of Surgery, Skåne University Hospital Malmö, Malmö, Sweden

## Abstract

**Background:**

The aim was to investigate whether adding a reinforced tension-line (RTL) suture to a standard 4 : 1 small-bite closure would reduce the incidence of incisional hernia after colorectal cancer surgery.

**Methods:**

Patients aged at least 18 years, who were scheduled for elective colorectal cancer surgery through a midline incision at two Swedish hospitals (2017–2021), were randomized in a 1 : 1 ratio to either fascial closure with RTL and 4 : 1 small-bite closure with polypropylene sutures (RTL group) or 4 : 1 small-bite closure with polydioxanone suture alone (PDS group). The primary outcome was CT-detected incisional hernia 1 year after surgery. CT interpreters were blinded regarding treatment group.

**Results:**

In all, 160 patients were randomized, 80 in each group. The study closed early to recruitment and follow-up. Some 134 patients were analysed at 1 year: 63 in the RTL group and 71 in the PDS group. Nineteen patients were found to have an incisional hernia: 4 (6%) in the RTL group and 15 (21%) in the PDS group (OR 3.95, 95% c.i. 1.24 to 12.60; *P* = 0.014). No unintended effects were found in either group.

**Conclusion:**

Adding an RTL suture at fascial closure decreased the incidence of incisional hernia in patients undergoing surgery for colorectal cancer. Trial registration: NCT03390764 (https://clinicaltrials.gov).

## Teaser

Incisional hernia is a common complication after laparotomy. An alternative to mesh reinforcement in fascial closure is the reinforced tension-line (RTL) suture technique. In this RCT of patients undergoing elective surgery for colorectal cancer, the RTL technique prevented incisional hernia compared with the 4 : 1 small-bite technique (*P* = 0.014).

## Introduction

Surgery is commonly performed for colorectal cancer. Even with minimally invasive surgery, open access for specimen extraction is needed. Incisional hernia is one of the most common complications after abdominal surgery and is a major cause of surgical morbidity. The reported incisional hernia incidence ranges between 0 and 36%^1^. Even when strictly standardized closure techniques are used, such as in the STITCH trial^2^ that compared large-bite with small-bite closure, the incidence was 21 and 13% respectively after 1 year.

Risk factors for incisional hernia include diabetes, overweight/obesity, smoking, immunosuppression, midline incision (*versus* off-midline incisions), closure technique other than the continuous small-bite technique, and surgical-site infection^3^. Patients with colorectal cancer often have several of these risk factors and are therefore at high risk of incisional hernia, with data from Sweden indicating an incidence of 26% on CT evaluation 1 year after tumour resection^4^. The 4 : 1 small-bite technique, described and evaluated by Milbourn *et al*.^5^, is considered the standard for fascial closure and is the technique recommended in the European Hernia Society (EHS) guidelines^6^. Despite use of this technique, incisional hernias still develop. Use of prophylactic mesh has been shown to decrease rates in patients at high risk^7,8^, but, because colorectal cancer surgery is mostly classified as clean-contaminated, concern exists about the risk of infection and increased costs associated with mesh use^9–11^.

The reinforced tension-line (RTL) suture technique was described by Hollinsky *et al.* in 2007^12^ as an alternative to mesh for treating incisional hernia. In their description, non-absorbable polypropylene sutures were used. Reports evaluating the RTL technique are sparse. Agarwal *et al.*^13^ used the technique successfully with slowly absorbable polydioxanone sutures for prevention of fascial dehiscence in laparotomies for acute peritonitis. A recent RCT^14^ on incisional hernia prevention, which compared RTL and 4 : 1 closure with 4 : 1 closure alone, showed a lower incidence of hernia with use of the RTL suture at 3-year follow-up (9.8 *versus* 28.3%). In that study, polydioxanone sutures were used in both groups and a large-bite technique was used for closure in the 4 : 1 group. In a local retrospective study^15^ of patients undergoing cytoreductive surgery and hyperthermic intraperitoneal chemotherapy (HIPEC) for peritoneal carcinomatosis, RTL suture compared favourably with 4 : 1 closure alone (2 *versus* 11%).

The present study investigated whether combining RTL suture with the 4 : 1 small-bite technique would result in fewer incisional hernias than standard 4 : 1 small-bite closure in patients undergoing elective surgery for colorectal cancer. The primary aim was to compare the incidence of CT-diagnosed incisional hernia at 1 year after surgery. Secondary aims included comparison of fascial closure time, incidence of surgical-site adverse events, overall complications, and clinical incisional hernia rate at 1 year.

## Methods

### Trial design

This open parallel RCT was conducted at Skåne University Hospital Malmö and the County Hospital Kristianstad, Sweden. It was designed to compare two fascial closure techniques regarding CT-diagnosed incisional hernia incidence 1 year after colorectal cancer surgery through a midline laparotomy.

Patients were assessed for eligibility and participation by the surgeon who scheduled the patient for operation, or by C.L.W., U.P. or P.R., the day before surgery. All patients signed informed consent to participate in the study. Inclusion and randomization were undertaken immediately before closing the fascia.

The study was approved by the Regional Ethics Committee at Lund University, Sweden (2017/459) and registered at https://clinicaltrials.gov (NCT03390764). The trial adhered to the recommendations of the Consolidated Standards of Reporting Trials.

### Participants

Patients aged at least 18 years who were scheduled for open surgery for colorectal cancer through a midline laparotomy between 2017 and 2021 were eligible for inclusion. Preoperative exclusion criteria were: planned cytoreductive surgery/HIPEC, previous midline hernia surgery, current midline hernia (umbilical hernia smaller than 1 cm was accepted), inability to participate in follow-up, and ASA fitness grade more than III. Patients with hernia detected at the time of operation, need for fascial reconstruction, or peritoneal carcinomatosis, and those deemed not suitable by the surgeons were also excluded. Only patients in whom CT was undertaken at 12 ± 3 months after surgery were included in the statistical analyses. Patients reoperated within 9 months for any reason were excluded.

### Interventions

All patients received infection and thrombosis prophylaxis according to routine protocols in the unit of operation. Fascial closure was performed by the colorectal team that carried out the colorectal resection.

**Fig. 1:**
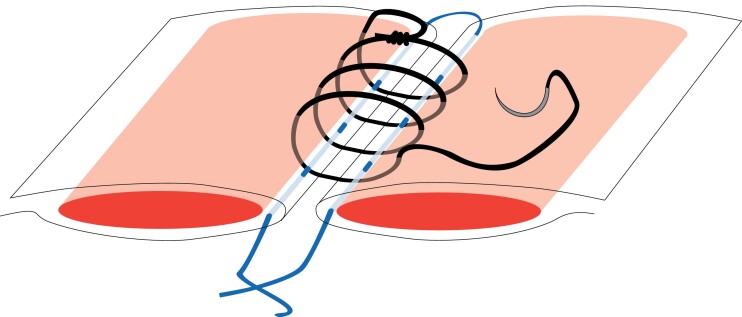
Schematic illustration of the combination of reinforced tension-line and 4 : 1 sutures The reinforced tension-line (RTL) suture (blue) is threaded within the linea alba along the incision on both sides. The 4 : 1 suture (black) is placed lateral to, and includes, the RTL suture in every stitch.

In the RTL group, a reinforcing suture of 2/0 polypropylene on a CT-2 needle (Prolene^®^; Ethicon, Raritan, NJ, USA) was applied along both sides of the incision within the condensed linea alba, as described by Hollinsky *et al.*^12^. The fascia was dissected free from subcutaneous fat 1 cm outside the incision in all directions, which included detaching the umbilicus from the fascia. The suture was started at one end of the incision and threaded within the fascia, parallel and 5–8 mm from the incision on both sides. The two suture ends were left untied. In the event of an opened rectus muscle sheath with exposed muscle, the RTL suture was used to close the fascial layers. Next, the incision was closed using the 4 : 1 small-bite technique according to Millbourn *et al.*^5^. This was done with the same type of suture placed just outside and including the RTL suture in every stitch and 5 mm apart. Mass closure including muscle was not intended. Finally, the RTL suture was tied ( *Fig. 1*).

In the PDS group, the incision was closed using the 4 : 1 small-bite technique^5^, with a 2/0 polydioxanone suture on a CT-2 needle (PDS^®^Plus; Ethicon). The suture was placed 5–8 mm from the fascial edges 5 mm apart, only including the fascia.

Skin closure was done with a running intracutaneous 4/0 polydioxanone suture (PDS^®^Plus) in both groups.

An instruction video presenting the closure techniques was produced by U.P. and C.L.W., and distributed to participating surgeons. Several study meetings were held with these surgeons, and unannounced operating room visits were undertaken by C.L.W. for inspection of how the closure techniques were carried out.

### Outcomes

The primary aim was to compare the CT-diagnosed incidence of incisional hernia at 12 ± 3 months after surgery between the study groups. One year after operation, patients underwent CT as part of the regular follow-up after colorectal cancer treatment, in the supine position without the Valsalva manoeuvre. CT images were scrutinized by three independent examiners (2 surgeons and 1 radiologist) who were blinded regarding the fascial closure technique. Any discrepancy in the assessment was discussed to reach consensus. Incisional hernia was defined according to the EHS^16^ as ‘any abdominal wall gap with or without a bulge in the area of a postoperative scar, perceptible or palpable by clinical examination or imaging’. A postoperative diastasis between the rectus abdominis muscles, without a concomitant gap, was not defined as an incisional hernia. A gap of less than 10 mm in the umbilicus, present before as well as after surgery, was not considered an incisional hernia.

Secondary aims were to compare the fascial closure time, accidental opening of the rectus sheath, incidence of surgical-site complications (haematoma, seroma, and surgical-site infection), overall 30-day complications according to the Clavien–Dindo classification^17^, and incisional hernia at physical examination after 1 year.

Patient data were registered prospectively according to study protocols applicable at inclusion, during the hospital stay, and at outpatient visits. At 1 month after operation, the patients were seen in the outpatient clinic by a colorectal surgeon. Any short-term complication was registered, and wound healing was assessed.

At 1 year after operation, patients were invited to a clinical examination. Those who responded were seen by a surgeon in the research group. Clinical examination was performed with the patient standing and in the supine position in a relaxed state and when straining and coughing.

During the COVID-19 pandemic, restrictions on visits to outpatient clinics were introduced, leading to cancelled or delayed study follow-up visits. Patients not attending the visits were contacted by telephone for an interview.

Clinical records were reviewed to identify events in between visits, and patients were asked to report any event that could be related to the operation.

### Sample size

The study was powered for the primary endpoint of CT-detected incisional hernia after 1 year and not any of the secondary endpoints. The incidence of incisional hernia after closure with the 4 : 1 small-bite technique was set to 20%, based on findings indicating a rate of 25% at 1 year in the authors’ department^4^. At this time, no publication on incisional herniation rates after use of RTL sutures was available and the rate was therefore assumed to be 5%. With a significance level of 0.05 and a power of 80%, 76 evaluable patients were needed in each group, based on a 2-tailed χ^2^ test. Assuming 20% drop-out, a minimum of 90 randomized patients was required in each group.

### Randomization

The randomization sequence was created using Microsoft^®^ Excel 365 for Windows^®^ (Microsoft, Redmond, WA, USA). The computer-generated list was used for allocation, and the group allocation was placed in sequentially numbered sealed opaque envelopes. Randomization was stratified only by study centre with a 1 : 1 distribution between study groups, and permuted block sizes of 4, 6 or 8.

### Blinding

Neither recruiting nor operating surgeons were involved in any part of the randomization sequence process, and they were not aware of the block sizes. The three independent CT image examiners were blinded regarding the fascial closure technique. The examining surgeon at the 1-year follow-up visits was blinded to the CT examination outcome.

### Statistical analysis

Continuous variables are expressed as mean(s.d.), and were evaluated with Student’s *t* test. For qualitative variables, Pearson’s χ^2^ test was used, or Fisher’s exact test if the number for any expected outcome was below 5. All tests were two-tailed. Risk was quantified by means of an OR with 95% confidence interval. Study results were analysed on an intention-to-treat basis. Study fragility index (number of non-events needed to turn into events in the group with least events until the statistically significant difference is lost)^18^ and number needed to treat were calculated. *P* ≤ 0.050 was considered statistically significant. All statistical analyses were carried out using SPSS^®^ version 26.0.0.1 (IBM, Armonk, NY, USA).

## Results

### Recruitment and participant flow

In total, 248 patients were assessed for inclusion. Trial inclusion was stopped when 204 patients (248 minus 25 patients who declined participation, and 19 who did not meet inclusion criteria) were scheduled for surgery. Owing to the pandemic, the organization of colorectal surgery in the Region of Skåne was changed, leading to referral of patients to hospitals other than the study centres. At the same time, laparoscopic operations for colorectal cancer increased dramatically. For these reasons, the inclusion rate slowed down tangibly, and inclusion in the study was stopped.

**Fig. 2:**
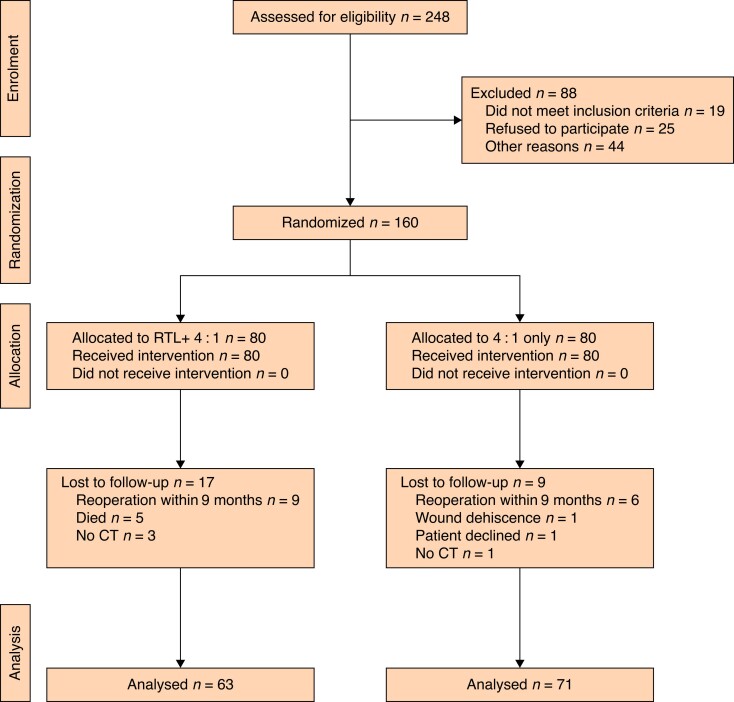
CONSORT diagram for the trial RTL, reinforced tension line.

Factors that resulted in peroperative exclusion were: surgeons decided not to randomize (17), hernia detected (9), fascial reconstruction needed (6), peritoneal carcinomatosis diagnosed (5), and other peroperative reasons for not randomizing (7). This resulted in 160 patients, 80 in each group, being randomized at operation and all received the allocated intervention. A total of 134 patients remained for analysis at 1 year ± 3 months: 63 in the RTL group and 71 in the PDS group (*Fig. 2*). The study reached a power of 75.1%, not the desired 80%.

Some 79 of 134 patients attended the 1-year clinical follow-up visit. Reasons for 55 patients not attending were: COVID 19 restrictions (27), poor health (9), declined owing to long journey (11), and declined for other reason (8).

### Baseline data, neoadjuvant therapy use, and perioperative outcomes

Demographic data for the included patients are presented in *Table 1*. Neoadjuvant therapy and perioperative data are shown in *Table 2*. A suture : wound length ratio of more than 4 was achieved in all but four patients, two in the RTL group (ratio 3.3 and 3.7) and two in the PDS group (ratio 3.5 and 3.8). The ratio was missing for four patients, two in each group. Fascial closure time was longer (42 *versus* 30 min; *P* < 0.001) and stoma creation was more frequent (*P* = 0.020) in the RTL group, as was receipt of neoadjuvant radiotherapy (*P* = 0.030).

**Table 1:** Patient baseline characteristics

	Total (*n* = 160)	RTL (*n* = 80)	PDS (*n* = 80)	Lost to follow-up at 1 year (*n* = 26)
Age (years), mean(s.d.) (95% c.i.)	68.4 (11.7) (66.5, 70.2)	70 (11) (67, 72)	67 (12) (64, 70)	68 (14) (62, 74)
**Sex**				
Female	74 (46.3; 38.6, 54.0)	38 (48; 37, 58)	36 (45.0; 34, 56)	15 (58; 39, 75)
Male	86 (53.8; 46.0, 61.4)	42 (52; 42, 63)	44 (55; 44, 66)	11 (42; 25, 61)
BMI (kg/m^2^), mean(s.d.) (95% c.i.)	25.6(4.3) (24.9, 26.3)	26(4.3) (25, 27)	25(4) (24, 26)	24 (4) (23, 26)
Previous midline incision	83 (51.9; 44.2, 59.5)	38 (48; 37, 58)	45 (56; 45, 67)	11 (42; 25, 61)
Cardiovascular disease	80 (50.0; 42.3, 57.7)	45 (56; 45, 67)	35 (44; 33, 55)	15 (58; 39, 75)
COPD	15 (9.4; 5.6, 14.6)	8 (10; 5, 18)	7 (9; 4, 16)	3 (12; 3, 28)
Diabetes mellitus	23 (14.4; 9.6, 20.4)	12 (15; 9, 24)	11 (14; 8, 23)	3 (12; 3, 28)
Steroid treatment	7 (4.4; 2.0, 8.4)	4 (5; 2, 11)	3 (4; 1, 10)	1 (4; 0, 17)
Other immunosuppression	3 (1.9; 0.5, 4.9)	1 (1; 0, 6)	2 (3; 1, 8)	1 (4; 0, 17)
**ASA fitness grade**				
I	13 (8.1; 4.6, 13.1)	6 (8; 3, 15)	7 (9; 4, 16)	0 (0; 0, 0)
II	93 (58.1; 50.4, 65.6)	42 (53; 42, 63)	51 (64; 53, 74)	10 (39; 22, 58)
III	54 (33.8; 26.8, 41.3)	32 (40; 30, 51)	22 (28; 19, 38)	16 (62; 42, 78)
Smoker	20 (12.5; 8.1, 18.3)	12 (15; 9, 24)	8 (10; 5, 18)	3 (12; 3, 28)
Haemoglobin (g/l), mean(s.d.) (95% c.i.)	123.7(13.4) (121.6, 125.8)	123 (14) (120, 127)	124 (12) (121, 127)	120 (13) (114, 125)
Albumin (g/l), mean(s.d.) (95% c.i.)	37.2(4.6) (36.4, 37.9)	37 (5) (36, 38)	37 (5) (36, 38)	35 (6) (33, 37)

Values are *n* (%, with 95% c.i.), unless otherwise indicated. COPD, chronic obstructive pulmonary disease. RTL, reinforced tension-line and 4 : 1 small-bite closure with polypropylene sutures; PDS, 4 : 1 small-bite closure with polydioxanone suture alone.

**Table 2:** Neoadjuvant treatment and perioperative outcomes

	Total (*n* = 134)	RTL (*n* = 63)	PDS (*n* = 71)	*P*
Neoadjuvant cytostatics	57 (42.5; 34.4, 51.0)	28 (44; 33, 57)	29 (41; 30, 53)	0.674
Neoadjuvant radiotherapy				0.030‡
None	77 (57.5; 49.0, 65.6)	30 (48; 36, 60)	47 (66; 55, 76)	
Short course	29 (21.6; 15.3, 29.2)	19 (30; 20, 42)	10 (14; 8, 24)	
Long course	28 (20.9; 14.7, 28.4)	14 (22; 13, 34)	14 (20; 12, 30)	
Suture : wound length ratio, mean(s.d.) (95% c.i.)	5.7 (1.5) (5.5, 6.0)	6 (1) (6, 6)	6(2) (5, 6)	0.549
Fascial closure time (min), mean(s.d.) (95% c.i.)	35 (14) (33, 38)	42 (15) (38, 45)	30 (11) (28, 33)	< 0.001
Duration of operation (min), mean(s.d.) (95% c.i.)	389 (161) (362, 416)	418 (152) (380, 456)	363 (165) (324, 402)	0.157
Blood loss (ml), mean(s.d.) (95% c.i.)	419 (412) (348, 489)	477 (368) (384, 571)	368 (443) (263, 473)	0.284
**Accidental opening of rectus muscle fascia**				
Above umbilicus	26 (20.2; 13.9, 27.7)	12 (20; 11, 31)	14 (21; 12, 31)	0.897
At umbilicus	31 (24.0; 17.3, 31.9)	16 (26; 17, 38)	15 (22; 14, 33)	0.580
Below umbilicus–arcuate line	19 (14.7; 9.4, 21.6)	8 (13; 6, 23)	11 (16; 9, 26)	0.624
Stoma creation	73 (54.5; 46.0, 62.7)	41 (65; 53, 76)	32 (45; 34, 57)	0.020
Stoma take-down	22 (16.4; 10.9, 23.4)	7 (11; 5, 21)	15 (21; 13, 32)	0.118
Perioperative complication	8 (6.0; 2.9, 10.9)	5 (8; 3, 17)	3 (4; 1, 11)	0.474

Values are *n* (%; 95% c.i.), unless otherwise indicated. RTL, reinforced tension-line and 4 : 1 small-bite closure with polypropylene sutures; PDS, 4 : 1 small-bite closure with polydioxanone suture alone. ‡No neoadjuvant radiotherapy *versus* short/long-course radiotherapy.

Of the surgical interventions performed, 41% were colonic resections and 59% rectal procedures, including pelvic exenteration in 6 patients and musculocutaneous flap reconstructions of perineal defects in 17. The surgical complexity is summarized in *Table 3*.

**Table 3:** Surgical complexity

	No. of patients
Patients available for 1-year evaluation	134 (100.0)
**Colonic resection**	55 (41.0)
Colonic resection only	43 (78.2)
Colonic resection and resection of other organ	12 (21.8)
**APR/rectal resection**	73 (54.5)
APR/rectal resection only	35 (47.9)
APR/rectal resection and gluteal flap	7 (9.6)
APR/rectal resection and resection of other organ	25 (34.2)
APR/rectal resection and resection of other organ and gluteal flap	6 (8.2)
**Pelvic exenteration**	6 (4.5)
Pelvic exenteration only	2 (33.3)
Pelvic exenteration and gluteal flap	3 (50.0)
Pelvic exenteration and resection of other organ and gluteal flap	1 (16.7)

Values are *n* (%). APR, abdominoperineal resection.

### Postoperative outcomes and incisional hernia incidence

Postoperative outcomes and incidence are shown in *Table 4*. One patient in the PDS group and none in the RTL group developed wound dehiscence. In addition to that patient, nine patients in the RTL group and six in the PDS group were reoperated within 9 months, three and two respectively during the index admission. Indications for reoperation in the RTL group were bowel obstruction (3), anastomotic leak (2), intra-abdominal abscess (1), haematoma (1), loop ileostomy closure converted to laparotomy (1), and liver metastasis (1). Indications for reoperation in the PDS group were bowel obstruction (2), anastomotic leaks (2), colonic ischaemia (1), and liver metastasis (1).

**Table 4:** Postoperative outcomes

	Total (*n* = 134)	RTL (*n* = 63)	PDS (*n* = 71)	*P*
Complications within 30 days	83 (61.9; 53.5, 69.8)	40 (64; 51, 75)	43 (61; 49, 71)	0.727
Postoperative paralysis for > 3 days	55 (41.0; 33.0, 49.5)	25 (40; 28, 52)	30 (42; 31, 54)	0.763
Pneumonia	2 (1.5; 0.3, 4.7)	0 (0; 0, 0)	2 (3; 1, 9)	0.498
Thromboembolism	3 (2.2; 0.6, 5.9)	1 (2; 0, 7)	2 (3; 1, 9)	1.000
Anastomotic insufficiency	3 (2.2; 0.6, 5.9)	1 (2; 0, 7)	2 (3; 1, 9)	1.000
Incisional haematoma	1 (0.7; 0.1, 3.4)	1 (2; 0, 7)	0 (0; 0, 0)	0.470
Incisional secretion/leakage	11 (8.2; 4.4, 13.8)	3 (5; 1, 12)	8 (11; 6, 20)	0.171
Seroma	8 (6.0; 2.9, 10.9)	3 (5; 1, 12)	5 (7; 3, 15)	0.722
**Wound infection**				0.914†
None	123 (91.8; 86.2, 95.6)	58 (92; 84, 97)	65 (92; 83, 96)	
Superficial	10 (7.5; 3.9, 12.8)	4 (6; 2, 14)	6 (9; 4, 17)	
Deep	1 (0.7; 0.1, 3.4)	1 (2; 0, 7)	0 (0; 0, 0)	
**Clavien–Dindo grade**				0.091‡
No complication	4 (3.0; 1.0, 6.9)	1 (2; 0, 7)	3 (4; 1, 11)	
I	47 (35.1; 27.4, 43.4)	20 (32; 21, 44)	27 (38; 27, 50)	
II	70 (52.2; 43.8, 60.6)	33 (52; 40, 64)	37 (52; 41, 64)	
IIIa	5 (3.7; 1.4, 8.0)	2 (3.; 1, 10)	3 (4; 1, 11)	
IIIb	4 (3.0; 1.0, 6.9)	3 (5; 1, 12)	1 (1; 0, 6)	
IVa	4 (3.0; 1.0, 6.9)	4 (6; 2, 14)	0 (0; 0, 0)	
Adjuvant treatment	56 (42.1; 34.0, 50.6)	22 (35; 24, 47)	34 (49; 37, 60)	0.111
Wound healed at 1 month	115 (88.5; 82.1, 93.1)	52 (87; 76, 94)	63 (90; 81, 95)	0.553
CT-verified hernia at 1 year	19 (14.2; 9.1, 20.8)	4 (6; 2, 14)	15 (21; 13, 32)	0.014

Values are *n* (%; 95% c.i.). RTL, reinforced tension-line and 4 : 1 small-bite closure with polypropylene sutures; PDS, 4 : 1 small-bite closure with polydioxanone suture alone. †No wound infection *versus* superficial/deep wound infection. ‡No complication or grade I, II *versus* IIIa, V (need for interventions, ICU treatment or death).

There were five deaths during the first postoperative year, all in the RTL group (2 disseminated cancer, 1 bowel ischaemia, 1 ruptured abdominal aortic aneurysm, and 1 chronic lymphatic leukaemia and old age).

CT examination at 1 year revealed incisional herniation in 19 patients: 4 (6%) in the RTL group and 15 (21%) in the PDS group, with a risk difference of 14.7% (OR 3.95, 95% c.i. 1.24 to 12.60; *P* = 0.014). Among these, one patient with a suture : wound length ratio below 4 and one patient without data on this variable, both in the PDS group, had an incisional hernia. The width of the hernia was less than 4 cm in 4 patients, 4–10 cm in 13, and over 10 cm in 2. The rate did not differ between operating centres (OR 1.08, 0.28 to 4.11; *P* = 1.000).

The fragility index of the study was 2, corresponding to 50% of the incisional hernias in the RTL group. The number needed to treat was 6.8. Of the 19 patients with incisional hernia, 12 attended the 1-year follow-up visit, and 2 of the 12 incisional hernias were missed by clinical examination. Of those with incisional hernia, only one patient in the PDS group had been operated for incisional hernia shortly after the 1-year CT examination.

### Adverse events

Rates of complication and unintended effects were similar in the two groups.

## Discussion

This two-centre RCT compared incisional hernia rates between RTL suture in combination with the 4 : 1 small-bite technique and the 4 : 1 small-bite technique alone after midline laparotomy for colorectal cancer in an elective setting. For the primary endpoint, incisional hernia rate at CT examination after 1 year, a reduction in incidence favouring the RTL group was found, without any differences in complications.

The RTL suture has been evaluated sparsely. One study^14^ designed to evaluate the RTL technique for prevention of incisional herniation, which included both elective and acute patients with a high risk of fascial dehiscence and incisional hernia development, reported a hernia incidence of 9.8% with RTL and 4 : 1 closure *versus* 28.3% for 4 : 1 closure alone at 3-year follow-up. The corresponding figures 1 year after elective colorectal cancer surgery in the present study were similar. The incidence of incisional hernia is known to increase over time, so a higher incidence with longer follow-up must also be anticipated in the present study. Whether the difference between groups persists or not over time remains to be evaluated.

Polypropylene suture was used in the RTL group in the present study. Polydioxanone was chosen for the group with closure using the 4 : 1 technique alone because this is the authors’ standard closure technique. It could be argued that an absorbable suture should have been used for reinforcement, but the intention of using polypropylene was that it remained as a permanent reinforcement.

CT has a higher sensitivity than clinical examination for detecting fascial gaps and is routinely performed after surgery for colorectal cancer^19^; this is why it was chosen for diagnosis of incisional hernia in the present study. The higher sensitivity for CT was also shown in this study, in which 17% of incisional hernias (2 of 12) detected by CT were missed by clinical examination. Arguably, some CT-detected incisional hernias were small and of minor clinical importance. However, the majority of the incisional hernias were CT-wise evident, which reduces the risk of diagnosing clinically unimportant fascial gaps.

Reaching the stipulated ratio of 4 : 1 is crucial in a study investigating closure techniques based on that ratio. Participating surgeons did so in all but four procedures, divided equally between the groups. Ratio data were missing for a further four procedures. The risk that missing data could have affected the result is deemed negligible because they were equally distributed between groups. The overall mean ratio of 5.7 indicates good adherence to the small-bite technique^20^. When applying the RTL suture, the fascia needs to be dissected around the incision. This, or the use of permanent sutures in the RTL group, did not lead to an increased rate of surgical-site complications, and the wound infection rate was in the expected range^21^. Applying the RTL suture extended the fascial closure time, but only by 12 min, which, combined with a relatively small number needed to treat of 6.8, should motivate routine adoption of this technique.

Twenty-six patients were lost to follow-up during the first postoperative year. Although this is not ideal methodologically, it reflects the real-world challenges of longer-term follow-up in RCTs. Five patients died, all in the RTL group, but no death was associated with the fascial closure technique. The same applied to the patients who were reoperated in the first year. The only reoperation associated with fascial closure was that for fascial dehiscence occurring in a patient in the PDS group.

This study has weaknesses. It was launched during a time when laparoscopic colorectal surgery increased dramatically in the authors’ region, and organizational changes introduced in 2020 to cope with the pandemic led to a decrease in colorectal operations at the study centres. If the dramatic increase in minimally invasive surgery for colorectal procedures had been anticipated, more participating centres would have been desirable, thereby possibly avoiding the observed reduction in pace of recruitment in the later stages of the trial, which led to the conclusion that recruitment should be stopped. At that time, 204 patients had been scheduled for surgery. Perioperative findings and the surgeons’ decision not to randomize resulted in a perioperative drop-out rate that exceeded expectations. Instead of the planned 152 evaluable patients, the final number was 134, yielding a study power of 75.1%, using the original study design assumptions. No risk factors for incisional hernia were stratified for in the randomization process. The difference in incidence of neoadjuvant radiotherapy between groups is most likely a random finding. As the incidence of neoadjuvant radiotherapy was higher in the RTL group, it seems unlikely that this influenced the results. Furthermore, no power calculations were undertaken for secondary endpoints, leading to some uncertainty concerning the statistical significance of these results. The pandemic enforced restrictions on study follow-up in the authors’ region. Furthermore, a long travel distance to study follow-up visits, for patients referred for surgery from other regions, decreased willingness to attend. These factors culminated in a reduction in the number of patients who were evaluated clinically at 1 year.

The strengths of the study are the RCT design, few missing data, with the exception of clinical 1-year follow-up data, and good adherence to the protocol. This study involved patients undergoing elective colorectal cancer surgery through a midline incision. Many operations for a variety of indications are done through a midline laparotomy incision, in both elective and emergency settings. Data from the present trial suggest that adding an RTL suture to the fascial closure might reduce incisional herniation rates in other settings, and future trials should look to evaluate this.

## Supplementary Material

znae265_Supplementary_Data

## Data Availability

Study data are available on request.
