# Supplementary material for: Reinforced tension-line suture after laparotomy: early results of the Rein4CeTo1 randomized clinical trial
Source: Br J Surg. 2024 Oct 30;111(10):znae265. doi: 10.1093/bjs/znae265 (PMC11523493; doi:10.1093/bjs/znae265)
Supplement: znae265_Supplementary_Data [file znae265_supplementary_data.zip › Supplementary_Material (protocol).docx]

# Study protocol for the Rein4ceTo1 study

# Incisional hernia after colorectal cancer surgery- a randomized controlled trial comparing the 4:1 technique with and without reinforcement suture in abdominal closure.

## Authors

Charlotta L Wenzelberg, MD, Department of Clinical Sciences Malmö, Lund University, and Department of Surgery, Skåne University Hospital Malmö, Sweden.

Ulf Petersson, MD Associate professor, Department of Clinical Sciences Malmö, Lund University, and Department of Surgery, Skåne University Hospital Malmö, Sweden.

## Corresponding author

Charlotta L Wenzelberg

Email: charlotta.larsson_wenzelberg@med.lu.se

Adress: Skånes Universitetssjukhus, Jan Waldenströmsgatan 11A, 205 02 Malmö

ORCID ID: 0000-0001-8079-1276

## Background

Incisional hernia (IH) is the most common long-term complication after abdominal surgery resulting in increased morbidity and reduced quality of life (1), as well as increased social costs. A recently published systematic review and meta-regression analysis of more than 14,500 patients reported an IH prevalence of 12.8% (2). In patients with increased risk of IH development (obesity, abdominal aortic aneurysm surgery, increasing age, male sex, diabetes mellitus, chronic obstructive pulmonary disease, anaemia, malnutrition/cachexia, smoking and medication with corticosteroids), incidences exceeding 35% have been reported (3).

A small stich abdominal closure technique with a continuous slowly absorbable suture with a suture: wound length (SL:WL) ratio of at least 4:1 has become the gold standard for closing midline incisions (4, 5). The standardized use of this technique in two randomized controlled trials resulted in an IH incidence of 5.6% and 13% at one year, respectively (4, 5). IH development continues over time and according to an analysis of the results of two prospective studies, which examined IH after one and three years respectively, a 60% increase in the incidence of IH can be expected between one and three years postoperatively (12.6% and 22.4%, respectively) (6). With the aim of reducing the IH incidence in patients at increased risk, reinforcing mesh has been used in abdominal closure with good results (7). With the use of mesh reinforcement, incidences of IH two years postoperatively between 0% (8) and 11.3% (9) have been reported. Because of the fear of mesh infection, many surgeons hesitate to use mesh prophylactically in clean-contaminated or contaminated surgical situations (10). Whether this fear of mesh infection is justified or not remains to be proven. Elective colorectal surgery is mostly classified as "clean-contaminated", and IH development can be as high as 35% when diagnosed by computed tomography (11). The use of reinforcing mesh at the abdominal wall closure for this group of patients has recently been suggested (12). Although the risk of mesh infection appears low, the costs of mesh use increase as does the risk of other mesh-specific complications. For these reasons, today mesh reinforcement as hernia prophylaxis is only recommended for high-risk patients (7). An alternative to mesh reinforcement as IH prophylaxis is therefore desirable.

A technique with suture reinforcement (Reinforced Tension Line) was described by Hollinsky in 2007 as a technique for IH operations, with good results (13). The technique has also been studied as a means of preventing fascial ruptures in acute abdominal surgery patients. In the majority of cases the surgery was for peritonitis, with improved results compared to standard suturing (14). The RTL technique is easy to perform, inexpensive, and could be an option to reinforce the abdominal wall closure without added cost or risk of complications. If so, the technique could be beneficial not only for patients undergoing surgery for colorectal cancer but also for other groups at increased risk of developing a hernia.

## Aim

The primary aim of this randomized controlled trial is to compare RTL-reinforced abdominal closure with standardized 4:1 small stich technique with standardized 4:1 small stich technique only for abdominal closure, regarding IH incidence one year after elective colorectal cancer surgery. Secondary objectives are to evaluate and compare the incidence of wound ruptures, wound complications, IH incidence and IH surgery after three years, and patient satisfaction.

## Materials and methods

*Study design and setting:* The study is a multi-centre prospective randomized controlled trial with two groups where fascial closure is performed using either the standardized 4:1 technique or this in combination with a reinforced tension line suture (the RTL group). In the group without RTL suture, slowly resorbable 2-0 PDS Plus is used on CT2 needle in accordance with the current standard for abdominal closure (the PDS group). In the RTL group, nonabsorbable 2-0 Prolene on CT2 needle is used to mimic mesh-reinforced abdominal closure. The study will be carried out at the surgical clinics at Skåne University Hospital Malmö (SUS), Central Hospital in Kristianstad and the Hospital in Ystad.

*Participants:* Eligible are patients, 18 years of age and older, scheduled for elective midline open colorectal surgery for colorectal cancer. The patients are included after being given oral and written information about the study and after signing informed consent. Exclusion criteria: presence of a hernia or previous midline IH surgery; ASA score >3; patients scheduled for peritonealectomy and HIPEC; patients who are unable to participate in a study for follow-up due to mental status or substance abuse.

*Definitions*

Wound classification: according to "the Centers for Disease Control and prevention (CDC)" in the CDC guidelines for the prevention of surgical wound infections (10). IH: according to the European Hernia Society definition (15, 16). Postoperative complications: according to Clavien-Dindo (17).

*Surgical technique/interventions*

In the RTL-group a reinforcing suture of 2-0 polypropylene on CT-2 needle (Prolene®Ethicon, Raritan, New Jersey, USA) is applied along both sides of the incision within the condensed linea alba according to Hollinsky (13). The fascia is dissected free from subcutaneous fat 1 cm outside the incision in all directions, which included detaching the umbilicus from the fascia. The suture is started at one end of the incision and threaded within the fascia, parallel and 5-8 mm from the incision on both sides. The two suture ends are left untied at this stage. In case of an opened rectus muscle sheath with exposed muscle, the RTL suture is used to close the fascial layers. Next the incision is closed using the 4:1 small-bite technique according to Millbourn (4). This is done with the same kind of suture placed just outside and including the RTL-suture in every stitch and 5 mm apart. Mass closure including muscle is not intended. Finally, the RTL suture is tied.

In the PDS-group the incision is closed using the 4:1 small-bite technique according to Millbourn (4), with a 2-0 polydioxanone suture on CT-2 needle (PDS®Plus, Ethicon, Raritan, New Jersey, USA). The suture is placed 5–8 mm from the fascial edges 5 mm apart, only including the fascia.

Skin closure is done with a running intracutaneous 4-0 polydioxanone suture by Ethicon (PDS®Plus, Ethicon, Raritan, New Jersey, USA) in both groups.

*Registered data*

Basic data such as gender, age, height, weight, smoking habits, other diseases and their treatments, previous operations, possible pre-treatment of the tumor disease before surgery, results of completed X-ray examinations and laboratory data from the time before the operation are collected. From the time of surgery, the following are recorded: operative measures in the abdominal wall, potential opening of the rectus fascia, time required for abdominal closure, length of thread used, length of the wound, use of antibiotics, possible complications and construction of a stoma. After the operation, the following are recorded: possible complications and their treatment, continued antibiotic use and length of stay. At the regular 1-month check-up at the reception, the patient is examined and then recorded: any additional treatment for the tumor disease, the stage of the tumor disease, any complications after discharge from the hospital, the healing of the wound and the presence of an IH in the abdominal wall. A specific study visit is carried out 1 and 3 years after the operation, whereby the following data are noted: weight, occurrence of new disease and possible treatment of this, presence of IH in the abdominal wall, results of carried out CT examinations (especially the CT examinations that are part of the care program for this type of tumor disease) as well as data concerning any further operations in the abdominal wall during the follow-up period. At these visits, the patients are asked to answer a questionnaire regarding abdominal wall symptoms (Ventral hernia pain questionnaire) and the EQ-5D-5L quality of life questionnaire.

*Clinical examination at follow-up*

Patients will be examined for IH and other abdominal wall-related symptoms. The examination for the detection of an IH will be carried out in both standing and supine positions in a relaxed state as well as during crouching and coughing.

*Questionnaires*

Abdominal wall complaint form: This is a modification of the validated “Ventral Hernia Pain Questionnaire” form, approved by the publishers (18). The questionnaire contains 22 questions designed to reflect patients' current pain experience, wound healing at follow-up, cosmetic or social limitations, and patient satisfaction with the outcome. If the patient suffers from pain, additional questions are added to characterize the pain in terms of frequency, duration, use of painkillers and impact on the patient's ability to perform daily activities. Quality of life form: EQ-5D-5L is used to describe patient-perceived quality of life after 1 and 3 years respectively.

*Statistics*

The study is powered for the primary endpoint CT-detected IH after 1 year. IH incidence after closure with the 4:1 small-bite technique is set to 20%, based on findings indicating 25% IH at 1 year in our department. At this time no publication on IH after use of RTL is available and is assumed to be 5%. With a significance level of 0.05 and a power of 80%, 76 evaluable patients were needed in each group, based on a two-tailed chi-square test. Assuming a 20% drop-out a minimum of 90 randomized patients were required in each group.

Statistical methods: Quantitative variables are expressed as median and interquartile range (IQR), and differences between groups are calculated with the Mann-Whitney U test or Student's t-test. Qualitative data are analysed with Pearson's χ2 test or Fisher's exact test. p≤ 0.05 is considered significant. Statistical analyses are performed with the Statistical Package for Social Sciences (SPSS) version 22 (SPSS, Chicago, IL, USA).

*Randomization procedure*

The randomization sequence is created using Microsoft Excel 365 for Windows. The computer-generated list is used for allocation, and the group allocation is placed in sequentially numbered sealed opaque envelopes. Randomization is stratified per study centre with 1:1 distribution between study groups and with permuted block sizes of 4, 6 or 8.

The randomization itself takes place intraoperatively when the abdominal closure is to be started.

*Data management*

For each patient, a study protocol is drawn up for the collection of data before, during and after the operation, which is then unidentified with regard to personal data and transferred to a database. Permits for the handling of personal data are applied for to the Personal Data Manager at Skane university hospital. Digitized code key for connection between personal data and serial number in the study is kept locked during the study period and then destroyed. Study data is managed on an external storage device without general availability or network connection. The main person in charge in Malmö is Ulf Petersson together with PhD student Charlotta Wenzelberg. Basic data that is normally available in the medical record systems is obtained from those to minimize disruption of the study in clinical everyday life.

*Ethical and other approval*

The study is approved by The Regional Ethics Committee at Lund University, Sweden (Dnr 2017/459) and registered at www.clinicaltrials.gov (NCT03390764). EuroQol gave permission for research use of EQ-5D-5L, ID:21776. The trial adheres to the recommendations of the Consolidated Standards of Reporting Trials (CONSORT).

## Importance

The technique has not previously been used to prevent IH. The technique is simple and inexpensive, and technique-related complications are not expected to occur. If it is possible to achieve the same IH prevention effect as with mesh reinforcement, without risking difficult-to-treat complications related to mesh use, then important knowledge has been obtained through this study. The technique is also applicable to other groups with an increased risk of hernia after abdominal surgery. Overall, if an advantage is shown, a large number of patients can benefit from the technique, with a reduced need for IH surgeries in the future. The care costs for treating complications after abdominal operations can then also be reduced.

## Publications

The results of the study are expected to be reported in three articles in international journals and will form the basis for Charlotta Wenzelberg's dissertation work. First publication will discuss the results up to one year and include the primary study objective of hernia at one year. The next article focuses on IH development at 3 years and risk factors for IH development within this patient cohort. The third article will focus on patient reported outcomes as abdominal wall problems and quality of life.

## References

1. van Ramshorst GH, Eker HH, Hop WC, Jeekel J, Lange JF (2012) Impact of incisional hernia on healthrelated quality of life and body image: a prospective cohort study. Am J Surg 204: 144–50. doi: 10.1016/j.amjsurg.2012.01.012
2. Bosanquet DC, Ansell J, Abdelrahman T, Cornish J, Harries R, Stimpson A, Davies L, Glasbey JCD, Frewer KA, Frewer NC, Russell D, Russell I, Torkington J (2015) Systematic review and meta-regression of factors affecting midline incisional hernia rates: analysis of 14 618 patients. PLoS ONE 10(9): e0138745. doi: 10.1371/journal.pone.0138745.
3. Bevis PM, Windhaber RA, Lear PA, Poskitt KR, Earnshaw JJ, Mitchell DC (2010) Randomized clinical trial of mesh versus sutured wound closure after open abdominal aortic aneurysm surgery. Br J Surg 97: 1497–502.
4. Millbourn D, Cengiz Y, Israelsson LA (2009) Effect of stitch length on wound complications after closure of midline incisions: a randomized controlled trial. Arch Surg 144(11):1056–1059.
5. Deerenberg EB, Harlaar JJ, Steyerberg EW, Lont HE, van Doorn HC, Heisterkamp J, Wijnhoven BP, Schouten WR, Cense HA, Stockmann HB, Berends FJ, Dijkhuizen FP, Dwarkasing RS, Jairam AP, van Ramshorst GH, Kleinrensink GJ, Jeekel J, Lange JF (2015) [Small bites versus large bites for closure of abdominal midline incisions (STITCH): a double-blind, multicentre, randomised controlled trial.](http://www.ncbi.nlm.nih.gov/pubmed/26188742) Lancet 386:1254-60. doi: 10.1016/S0140-6736(15)60459-7.
6. Fink C, Baumann P, Wente MN, Knebel P, Bruckner T, Ulrich A, Werner J, Büchler MW, Diener MK (2014) [Incisional hernia rate 3 years after midline laparotomy.](http://www.ncbi.nlm.nih.gov/pubmed/24281948) Br J Surg J 101(2):51-4. doi: 10.1002/bjs.9364.
7. Muysoms FE, Antoniou SA, Bury K, Campanelli G, Conze J, Cuccurullo D, de Beaux AC, Deerenberg EB, East B, Fortelny RH, Gillion JF, Henriksen NA, Israelsson L, Jairam A, Jänes A, Jeekel J, López-Cano M, Miserez M, Morales-Conde S, Sanders DL, Simons MP, Śmietański M, Venclauskas L, Berrevoet F; European Hernia Society (2015) [European Hernia Society guidelines on the closure of abdominal wall incisions.](http://www.ncbi.nlm.nih.gov/pubmed/25618025) Hernia 19(1):1-24. doi: 10.1007/s10029-014-1342-5.
8. Muysoms FE, Detry O, Vierendeels T, Huyghe M, Miserez M, Ruppert M, Tollens T, Defraigne JO, Berrevoet F (2016) [Prevention of Incisional Hernias by Prophylactic Mesh-augmented Reinforcement of Midline Laparotomies for Abdominal Aortic Aneurysm Treatment: A Randomized Controlled Trial.](http://www.ncbi.nlm.nih.gov/pubmed/26943336) Ann Surg 263(4):638-45. doi: 10.1097/SLA.0000000000001369
9. García-Ureña MÁ, López-Monclús J, Hernando LA, Montes DM, Valle de Lersundi AR, Pavón CC, Ceinos CJ, Quindós PL (2015) [Randomized controlled trial of the use of a large-pore polypropylene mesh to prevent incisional hernia in colorectal surgery.](http://www.ncbi.nlm.nih.gov/pubmed/25575254) Ann Surg 261(5):876-81. doi: 10.1097/SLA.0000000000001116
10. Garner JS (19869 CDC guideline for prevention of surgical wound infections, 1985. Supersedes guideline for prevention of surgical wound infections published in 1982. (Originally published in November 1985). Revised. Infect Control 7 (3):193-200
11. [Claes K](http://www.ncbi.nlm.nih.gov/pubmed/?term=Claes%20K%5BAuthor%5D&cauthor=true&cauthor_uid=24445348), [Beckers R](http://www.ncbi.nlm.nih.gov/pubmed/?term=Beckers%20R%5BAuthor%5D&cauthor=true&cauthor_uid=24445348), [Heindryckx E](http://www.ncbi.nlm.nih.gov/pubmed/?term=Heindryckx%20E%5BAuthor%5D&cauthor=true&cauthor_uid=24445348), [Kyle-Leinhase I](http://www.ncbi.nlm.nih.gov/pubmed/?term=Kyle-Leinhase%20I%5BAuthor%5D&cauthor=true&cauthor_uid=24445348), [Pletinckx P](http://www.ncbi.nlm.nih.gov/pubmed/?term=Pletinckx%20P%5BAuthor%5D&cauthor=true&cauthor_uid=24445348), [Claeys D](http://www.ncbi.nlm.nih.gov/pubmed/?term=Claeys%20D%5BAuthor%5D&cauthor=true&cauthor_uid=24445348), [Muysoms F](http://www.ncbi.nlm.nih.gov/pubmed/?term=Muysoms%20F%5BAuthor%5D&cauthor=true&cauthor_uid=24445348) (2014) Retrospective observational study on the incidence of incisional hernias after colorectal carcinoma resection with follow-up CT scan. Hernia 18(6):797-802. doi: 10.1007/s10029-014-1214-z.
12. Muysoms, F.E. & Dietz, U.A (2016). Prophylactic meshes in the abdominal wall. Chirurg doi:10.1007/s00104-016-0229-7. Published on-line:26 July 2016.
13. Hollinsky C, Sandberg S, Kocijan R (2007) Preliminary results with the reinforced tension line: a new technique for patients with ventral abdominal wall hernias. Am J Surg 194:234–239. doi:10.1016/j.amjsurg.2006.09.045
14. Agarwal A, Hossain Z, Agarwal A, Das A, Chakraborty S, Mitra N, Gupta M, Ray U (2011) [Reinforced tension line suture closure after midline laparotomy in emergency surgery.](http://www.ncbi.nlm.nih.gov/pubmed/21831931) Trop Doct 41(4):193-6. doi: 10.1258/td.2011.110045.
15. Korenkov M, Paul A, Sauerland S, Neugebauer E, Arndt M, Chevrel JP, Corcione F, Fingerhut A, Flament JB, Kux M, Matzinger A, Myrvold HE, Rath AM, Simmermacher RK (2001) Classification and surgical treatment of incisional hernia. Results of an experts’ meeting. Langenbecks Arch Surg 386:65-73. doi:10.1007/s004230000182-
16. Muses FE, Miserez M, Berrevoet F, Campanelli G, Champault GG, Chelala E, Dietz UA, Eker HH, El Nakadi I, Hauters P, Hidalgo Pascual M, Hoeferlin A, Klinge U, Montgomery A, Simmermacher RK, Simons MP, Smietański M, Sommeling C, Tollens T, Vierendeels T, Kingsnorth A (2009) Classification of primary and incisional abdominal wall hernias. Hernia 13:407-14. doi:10.1007/s10029-009-0518-x.
17. Dindo D, Demartines N, Clavien P-A (2004) Classification of surgical complications: a new proposal with evaluation in a cohort of 6336 patients and results of a survey. Ann Surg 240:205–213.
18. Clay L, Franneby U, Sandblom G, Gunnarsson U, Strigård K (2012) Validation of a questionnaire for the assessment of pain following ventral hernia repair--the VHPQ. Langenbecks Arch Surg 397:1219-24. doi:10.1007/s00423-012-0932-x.
